# What approaches exist to evaluate the effectiveness of UK-relevant natural flood management measures? A systematic map

**DOI:** 10.1186/s13750-023-00297-z

**Published:** 2023-05-23

**Authors:** Angela Connelly, Andrew Snow, Jeremy Carter, Jana Wendler, Rachel Lauwerijssen, Joseph Glentworth, Adam Barker, John Handley, Graham Haughton, James Rothwell

**Affiliations:** 1 Manchester School of Architecture, Manchester Metropolitan University, Manchestear, UK; 2 Department of Planning and Environmental Management, University of Manchester, Manchester, UK; 3 Berlin School for Public Engagement and Open Science, Berlin, Germany; 4 Department of Geography, University of Manchester, Manchester, UK

**Keywords:** Flooding, Flood risk management, Environmental Impact Assessment, Climate change adaptation, River catchments, Nature-based solutions, Natural Flood Management

## Abstract

**Background:**

This systematic map principally sought to understand the different forms of effectiveness that existing studies evaluate in relation to Natural Flood Management (NFM) in the UK with a supplementary question of whether studies engaged with climate change and future flood risk. NFM measures seek to protect, enhance, emulate, or restore the natural function of rivers as part of approaches to flood risk management (FRM). While there is agreement in both academic and practice/policy literature that NFM should be part of a holistic FRM strategy to address current and future flood risk, the specifics of how to expand the application of and consistently implement NFM successfully in practice are less well known. A core focus of this study is on how the effectiveness of NFM measures is evaluated in different studies based on approaches drawn from the Environmental Impact Assessment (EIA) literature: procedural, substantive, transactive and normative. The systematic map also examines how studies account for climate change, which is a crucial issue given the connections between NFM and climate change adaptation and resilience.

**Methods:**

We searched 13 bibliographic databases, Google scholar as a web-based search engine, and 21 organisational sites. Articles were screened by title, abstract, and full text based on defined eligibility criteria. Checks were performed for consistency amongst reviewers. Forms of effectiveness were coded on the basis of the included studies in the systematic map. The quantity and characteristics of the available evidence are summarised with the frequencies of effectiveness forms for each NFM measure are presented in heat maps.

**Review findings:**

A total of 216 articles reported eligible studies that were coded as part of the systematic map. Overall, the systematic map shows that the majority of studies considered at least one approach to effectiveness; however, very few studies considered multiple forms of effectiveness. The systematic map also demonstrates that climate change is considered systematically by around one-quarter of studies although many studies make claims about NFM’s effectiveness in the face of future climatic change.

**Conclusions:**

NFM can be effective in several different ways owing to their multiple benefits; however, there are evidence gaps around understanding these different forms of effectiveness. This is particularly marked for studies considering transactive and normative effectiveness. Interdisciplinary studies are more likely to consider multiple forms of effectiveness. This systematic map also found that whilst 75% of studies mention future climate change in their studies, only 24.1% contain a systematic consideration of the issue through, for example, using climate change projections. NFM is also at risk of climate change (e.g. through drought) and therefore it is imperative that study designs seek to incorporate consideration of effectiveness under future climate change. Policymakers should be made aware of the lack of understanding of how NFM measures perform under future climate change.

**Supplementary Information:**

The online version contains supplementary material available at 10.1186/s13750-023-00297-z.

## Background

Over the past decade, there has been increased interest in Natural Flood Management (NFM) measures that reduce flood risk through working with, instead of against, the natural environment. These measures include techniques such as land use management and river restoration that can be implemented to ‘help to protect, restore and emulate the natural functions of catchments, floodplains, rivers and the coast’ [1]. NFM works well at a catchment level where working with natural environment processes upstream can be used as part of a holistic flood risk management strategy that also encompasses downstream engineered flood defences [2]. Catchment-based flood risk management is a system-based approach that recognises the multiple sources of flood risk, accepting that while single measures to address coastal, pluvial, fluvial and groundwater flooding can work for a period, they neglect the hydrological system in its entirety. For the application of NFM, this conceptualisation necessitates the strategic application of both natural features and measures emulating natural function to support the interception, infiltration and storage of water and the naturalisation of channel flow [3].

The move towards natural flood management measures occurs in a context where the overall worth of nature-based solutions for helping us to adapt to climate change is being increasingly recognised by international organisations such as the Intergovernmental Panel on Climate Change (IPCC) [4]. The US Army Corps of Engineers have a high-profile initiative called ‘Engineering with Nature’ to align engineered with natural measures to reduce flood risk [5]. Across Europe, the EU Water Directive (2003) and the Floods Directive (2007) prompted moves towards integrated, catchment-based management which supported strategies that sought to work with natural processes and to achieve multiple benefits for society. The adoption of NFM within the UK as part of a holistic flood risk management strategy is thought to address numerous goals around climate change adaptation, biodiversity and health and well-being. Indeed, NFM is being piloted across the UK through the various environment agencies in the UK [6].

**Fig. 1:**
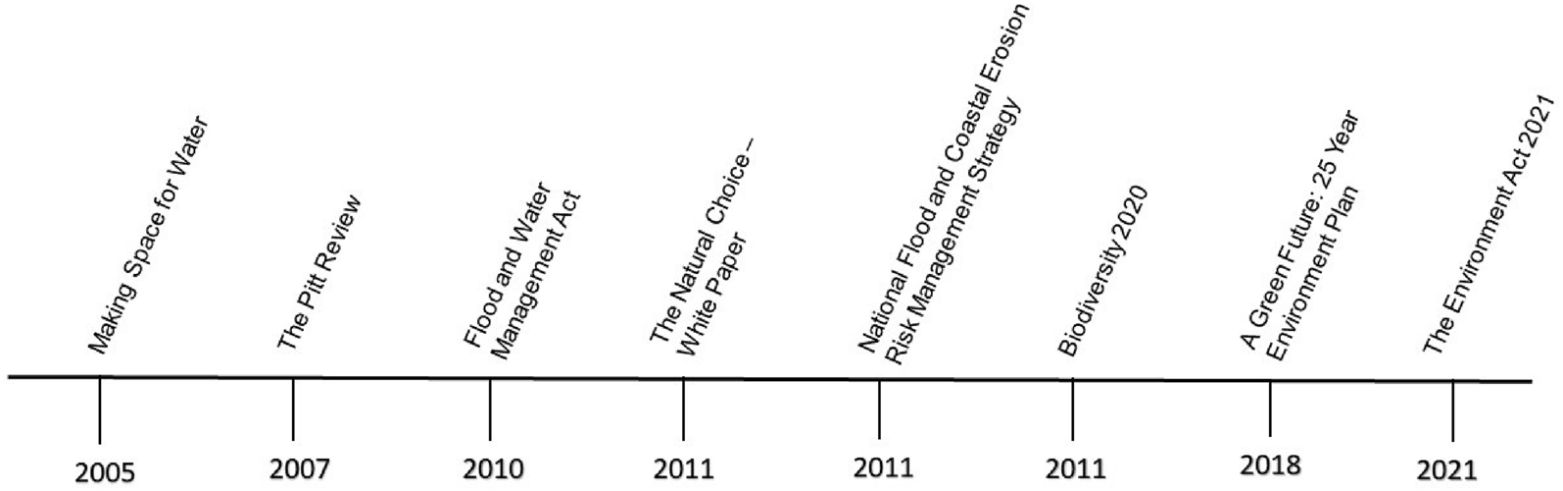
Policy timeline relating to natural flood management measures in England

In England, the NFM agenda has become more pronounced since 2004 following the Foresight *Future Flooding* project and the seminal strategy *Making Space for Water* (2005) which signalled the adoption of a new approach to flood risk management that sought to work with water rather than against it. England’s Environment Agency (EA) cite NFM as a way in which we can adapt to climate change [7]. In 2017, the English government allocated £15 million of funding to NFM projects and the approach is cemented in the 25-Year Plan (25 YEP) for the Environment which was published in 2018 The 25 YEP is now supported by the Environment Act which received Royal Assent in 2021. The UK’s devolved governments have also been at the forefront of developing NFM strategies; for example, the Scottish Environmental Protection Agency published its *Natural Flood Management Handbook* in 2015 [8] (Fig. 1).

With increasing attention being paid to NFM as an element of flood risk management, there is a need to understand more about the effectiveness of NFM measures, and how effectiveness is being perceived in this context. By effectiveness, we mean the extent to which NFM measures help to realise goals—not only in flood risk reduction but in terms of cost and benefits, increased participation and how such measures can help to realise wider ecological, economic and social benefits such as climate change adaptation, carbon sequestration, biodiversity restoration, improved water quality, and health and well-being [e.g. 3]. Policymakers and other implementers of NFM projects require a sound evidence base to give confidence to their decisions to invest in NFM which, in the UK, is typically based on cost–benefit analysis [9]. Consequently, much research is focussed on gathering underpinning evidence on the effectiveness of NFM from the perspective of capturing and slowing the flow of rainwater runoff. However, the range of NFM benefits means that there are a variety of ways that effectiveness can be framed and measured. Further, NFM measures will have to operate in the context of changing climatic conditions which may have a consequential effect on their effectiveness over longer timeframes and should therefore be factored into evaluations of NFM effectiveness.

There are several existing reviews that directly address or connect to the effectiveness of natural flood management measures, which are based on UK evidence or include evidence that is potentially relevant to the UK. Dadson et al. [10] focus on NFM effectiveness as measured in the natural sciences in terms of reducing flood frequency and hazard. However, NFM can realise multiple benefits beyond flood risk reduction. A wider view of these benefits was taken through an ecosystem adaptation perspective examined in a review by Iacob et al. [11]; however, the review only included a sample of 25 catchment-based projects from the UK, Europe and New Zealand rather than a systematic evidence review. Burgess-Gamble et al. [12] produced *Working with Natural Processes* for England’s Environment Agency sought to synthesise UK NFM practice and evidence. The literature review covered evidence from both the academic and grey literature; both nationally and internationally with evidence on the effectiveness of measures identified from the existing knowledge of those involved in the project. Burgess-Gamble et al. [12] mention studies that take account of climate change but future climate was not systematically considered in this review. Further, the focus was largely on UK-only NFM measures with case studies that try to look at the multiple benefits of NFM as well as providing insights into implementation issues. Given that NFM is also widely adopted in countries with a similar climate and regulatory background to the UK, such as the Netherlands, there is value in assessing evidence from other UK-relevant countries (in terms of their climate conditions and policy frameworks).

There is, consequently, an emerging evidence base on NFM and examples of synthesis of that evidence base. However, existing reviews often consider effectiveness a particular disciplinary perspective, e.g. the natural science base [10]. As noted above, NFM benefits, and goals of implementation, may be much wider than simply realising stated outcomes around capturing and ‘slowing the flow’ of rainwater and hence flood risk reduction. Wingfield et al. [3] point out that: ‘research and resources should be expanded beyond a principles, evidence and efficacy debate to mechanisms of NFM delivery’. This necessitates social scientific analysis and evidence around the framing of policy and, in particular, the processes, opportunities and barriers around NFM implementation in order to develop more politically effective responses to flooding. Both the policy and academic communities recognise that many policy issues in the environmental arena require multiple perspectives that are interdisciplinary and/or cover both the natural and social sciences [13]. Further, there has been a move towards large multi-disciplinary projects within the NFM agenda and, indeed, environmental management more broadly [e.g. 14]. However, it is not clear how the issue of measuring the effectiveness of NFM is being approached, and indeed how NFM effectiveness is being conceived, particularly when different disciplines are included in evidence reviews.

Several other issues must also be considered when assessing the effectiveness of NFM, whether that be in terms of evidence on outcomes or processes. Firstly, there is an issue around causality or the ‘attribution gap’ [15]. In many cases, a given catchment will need a variety of measures to effectively respond to the risk of flooding, and so it is difficult to isolate the effect of one NFM measure. Indeed, NFM will generally be one of a number of types of flood risk management measures, including traditional ‘hard’ engineered flood defences. In addition, the magnitude of a flood will influence the effectiveness of a given NFM measure [10]. Secondly, many studies are based on a short period of observation. However, the effectiveness of NFM may only be apparent over the long term—there may be a significant time lag between implementation and measurable impacts occurring, particularly if the objectives are around achieving multiple benefits such as improved health and well-being [16]. Consequently, this brings climate change into the picture given that organisations champion NFM partly as a climate change adaptation response [7]. Thirdly, the context and scale of implementation matter when considering NFM, and other nature-based solutions more broadly [3, 16]. Different environmental, economic, social and regulatory factors impact upon the implementation of an NFM particularly because they may be influenced by a wider range of variables than engineered structural defences [17]. Moreover, whilst catchment scale is where NFM should ideally be conceived and implemented, much of the evidence on NFM effectiveness occurs at local scales only [10, 18]. Similarly, the current political institutional context within England, for example, means that development and implementation of NFM is often localised [3]. Finally, there are particular issues to acknowledge when assessing NFM effectiveness taking account of future climate change. Climate data is modelled and so the perceived effectiveness of measures under future climate change will be projected rather than observed. It is not possible to be confident about the climatic and socio-economic conditions under which adaptation measures will operate, as these factors will evolve over time [19]. There may be some inconsistency in the findings of effectiveness studies which take account of climatic change. Moreover, the uncertainty may impact on the performance of measures in practice, which may be better or worse than anticipated. Such issues with climate change raises issues around how particular studies are addressing this shifting landscape through the use of climate change projections and/or models showing carbon sequestration potential.

Acknowledging these challenges, rather than trying to evaluate the effectiveness of NFM by comparing studies between one another (and trying to understand levels of confidence in effectiveness), we look at how existing studies approach and measure effectiveness following an approach detailed in the ‘Objective of the Review’ section. The systematic map also examines the scale at which a study takes place, the disciplinary focus of the study, and whether effectiveness concerning current and/or future flood risk under a changing climate is considered.

This systematic map builds on previous NFM work but, significantly, departs from that work in three main ways that will complement ongoing and future attempts to assess NFM evidence. Firstly, we explore the approaches taken to assessing NFM effectiveness from a broad perspective in order to move beyond the evidence and efficacy debate highlighted by Wingfield et al. [3]. Secondly, the focus on mapping approaches to NFM evidence means that we will consider published works from a variety of different disciplines and, indeed, interdisciplinary work that may approach evidence gathering and effectiveness in different ways. Lastly, we broadened the time and spatial horizon of the mapping by considering the extent to which climate change is taken account of when assessing evidence and by focusing on the inclusion of sources with a similar regulatory and climatic context to the UK. In doing so, we will provide a systematic map that will inform planning, policy-making and research around discussions related to the framing and measurement of the effectiveness of NFM. We also aim to utilise the review to address issues such as how climate change, and its accompanying uncertainty, may be addressed in future studies on NFM effectiveness within different disciplines.

### Stakeholder engagement

Discussions over the formulation of the question took place with members of the advisory group for the Natural Environment Research Council (NERC) *Environmental Evidence for the Future* initiative, further input from representatives of the UK Environment Agencies (including the Scottish Environment Protection Agency [SEPA] and Natural Resource Wales) and the Department for Environment, Food and Rural Affairs (Defra). In discussion with members of the Collaboration for Environmental Evidence (CEE), for example, the decision was taken to remove ‘comparator’ as a review question. The main research team also assembled an advisory group composed of four academics from disciplines including physical geography (with expertise in environmental pollution/environmental hydrology (n = 1), restoration ecology (n = 1), and planning and environmental management (with expertise on flood risk management policy) (n = 2) who helped to shape the review question, search strategy, and coding focus.

### Objective of the review

The main research question for this evidence map is: what approaches exist to evaluate the effectiveness of UK-relevant natural flood management measures?

A sub-question for the systematic evidence map is whether sources address climate change (i.e. future flood risk) in their study design. The identified sub-question builds on the expected analysis as detailed in an earlier systematic map protocol [20].

The review question has the following key elements:

- Population: Areas in the UK, or areas relevant to the UK, that are susceptible to current and/or future flood risk.
- Intervention: Specific, single NFM measures (listed in Table 1).
- Comparator: No comparator necessary.
- Outcome: Evaluation of the impact of NFM measure on current and/or future flood risk, Impact of biophysical, social, and/or political conditions on NFM and vice versa.

**Table 1:** Categories of measures within NFM approaches [1]

Rivers and floodplain management	Woodland management
River restoration	Catchment woodlands
Floodplain restoration	Floodplain woodlands
Leaky barriers	Riparian woodlands
Offline storage areas	Cross-slope woodlands

Run-off management	Coast and estuary management
Soil and land management	Saltmarsh and mudflats
Headwater drainage	Sand dunes
Run-off pathway management	Beach nourishment

The PICO elements of the review question contain no comparator because we aimed to systematically map the *forms* of effectiveness that different sources evaluate rather than seeking to understand the effect of different NFM measures against, for example, no intervention or an alternative intervention [see, e.g. 21]. The review covered peer-reviewed and grey literature of studies that have evaluated the effectiveness of NFM measures dependent on a range of outcomes.

Whilst we recognise that there are multiple definitions of the umbrella term ‘NFM’, we followed England’s Environment Agency (EA) definition of natural flood management which is ‘implementing measures that help to protect, restore and emulate the natural functions of catchments, floodplains, rivers and the coast’ [1]. The measures included in this definition are shown in Table 1, and it should be noted that this definition excludes measures such as Sustainable Urban Drainage Systems (SuDS) and other urban green infrastructure that seeks to slow down infiltration rates, such as street trees. Included within SuDS are features such as permeable paving, tend to be hard physical measures that do not ‘emulate the natural functions of catchments, floodplains, rivers and the coast’. We recognise that these terms can be difficult to characterise and the differences between the categories may be contested. The EA’s approach to Woodland Management, for example, captures the scale and location of different woodland types.

All types of flooding (fluvial, pluvial, coastal, groundwater) are included in the systematic map to cover the full gamut of NFM initiatives. The systematic map addresses issues including the different forms of effectiveness of NFM and the extent to which climate change and future flood risk is accounted for.

**Fig. 2:**
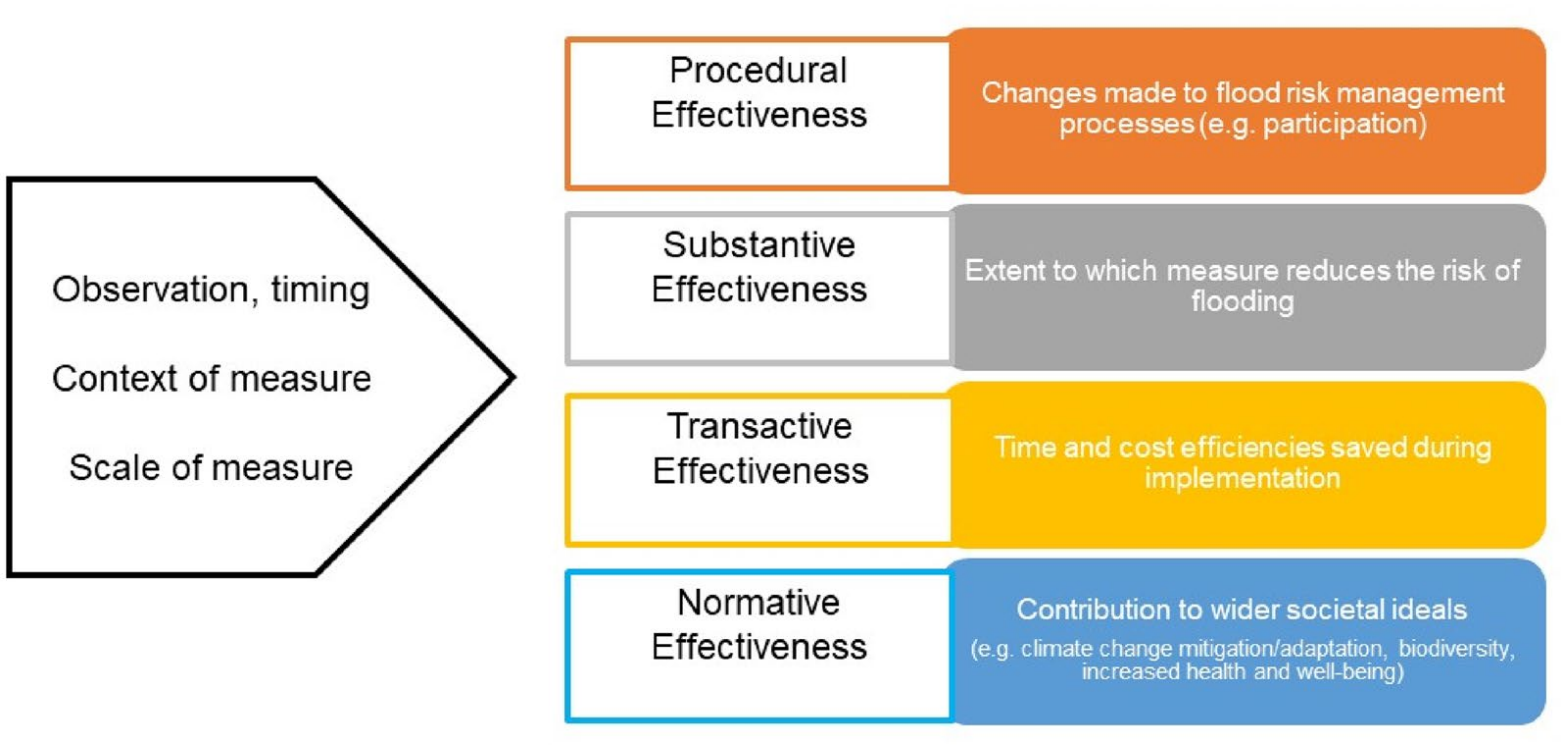
Model showing the identified forms of approaching effectiveness as a basis for coding articles

Drawing on the impact assessment (IA) literature, we identified different forms of effectiveness to provide a basis for conceptualising the multiple ways that NFM effectiveness can be evaluated [22]. Within IA, the ‘effectiveness’ forms relate to the focus of measurement where there is; (a) adherence to standardised processes (procedural effectiveness), (b) a contribution to a clearly defined, development-specific goal (substantive effectiveness), (c) time and cost savings that exceed those related to the application of IA (transactive effectiveness), and (d) some form of contribution to broader ideals such as sustainability (normative effectiveness) [23]. Building from this, and seeking to contribute to the literature on understanding NFM effectiveness, we have adopted the ‘forms’ of effectiveness used in the IA literature. Such a move helps to account for the observation that NFM not only seeks to reduce flood risk through lowering the volume of water reaching a receptor (or ‘substantive’ effectiveness) but demands new forms of practices, governance and decision-making (i.e. procedural effectiveness).In addition, NFM can be considered on the basis of time and cost efficiency (i.e. transactive effectiveness). NFM is primarily promoted because of the ability of projects to enable multiple objectives to be realised, e.g. biodiversity and increased health and well-being; so contributing to more sustainable forms of flood risk management (i.e. normative effectiveness). This can be seen in Table 2 and diagrammatically in Fig. 2. To this end, we incorporated effectiveness into our coding strategy based on the forms of effectiveness that included sources considered (see Additional file 1).

**Table 2:** Identified forms of effectiveness, their measurement and relevance to NFM

Form of effectiveness	What does this measure?	Relevance to NFM
Procedural	Adherence to standardised processes and best practices	NFM is argued to work best if governance frameworks are attuned to collaboration between diverse actors and knowledges Development of NFM should be context-sensitive—should not take a ‘cookie cutter’ approach Decision-making processes need to be altered so that FRM is addressed at a catchment system level NFM processes should take account of contextually relevant best practices
Substantive	Achievement of stated goal	NFM should contribute to the reduction of flood risk and this should be measurable
Transactive	Time and costs associated with the activity	The time and costs associated with NFM implementation (and maintenance where relevant) need to be made clear to enable cross-comparison NFM can potentially serve to reduce longer-term costs associated with climate change, particularly if urban development patterns are more sensitive to water as a result, but these savings must be calculated in a way that recognises inherent uncertainties with this type of projection
Normative	Contribution to broader ideals	NFM—if performed well—can contribute to broader ideals such as climate change mitigation, sustainability, health & wellbeing, and socio-ecological resilience, as well as increasing local cultural value and biodiversity Within this review we focus specifically on the normative benefits of NFM that relate to climate change mitigation and adaptation

Whilst we recognise that policy goals may change over time, the purpose of the review was not to assess the nature and quality of how a study examined effectiveness; rather, the focus was on simply isolating the forms of effectiveness that a study examined.

This study targets sources originating from inside and outside of the UK. There was insufficient time to include all NFM literature sources globally, and a research justification oriented around focusing the scope of the study on sources that are UK-relevant. The key issue here was to ensure a focus on UK-relevant studies so that the systematic map produced an output that is of value to UK planners and decision makers engaged in NFM activity. Climate-relevance is used within this study as a key criterion for including and excluding sources of evidence from beyond the UK. Here, the Koppen classification is used to identify UK-relevant sources based on the climate zone that they fall within [24]. Within this study, the focus is on European Union countries and regions falling within the UK’s Koppen Classification type (defined as warm temperate, fully humid, warm summer). This ensured that the sources come from locations that share the UK’s climate type, but that they are also covered by a similar regulatory regime concerning legislation such as the European Floods Directive. Other countries and regions from outside of the European Union also fall into this climate classification type, including New Zealand, South Eastern Australia, Serbia, South West Chile and the Pacific coast of North America, and literature sources from these countries are excluded due to different regulatory context.

Any approach to excluding literature sources addressing NFM measures will have its deficiencies, and climate-relevance is no exception. Indeed, the climate within Northern and Western Europe, which contains most of the UK’s climate-relevant countries and regions, varies significantly at finer levels of granularity. Climate was considered alongside a range of other types of exclusionary criteria with our advisory group of researchers specialising in topics linked to NFM, and the decision was taken to focus on climate as a key exclusionary criteria. Here, the perspective is taken that sources of NFM evidence originating from European Union countries and regions falling within the UK’s Koppen Classification type are of particular relevance to the UK because of their climate similarity and the resulting influence of the climate on flood risk and NFM. The decision was taken to focus on sources of literature emerging from the UK and countries within the European Union that share the UK’s *current* climate type, according to the Koppen Climate Classification, as opposed to future analogues. Future climate change is uncertain and multiple different future climate scenarios are therefore produced [25]. We do not know with any certainty which countries represent realistic future climate analogues for the UK, and this approach was therefore avoided.

## Methods

The protocol was published in Environmental Evidence [20]. This section outlines the approach and describes the major departures from the original protocol under the relevant sub-section. The protocol was developed in accordance with the RepOrting standards for Systematic Evidence Syntheses (ROSES) for systematic map [26] (Additional file 2). In addition, the systematic map followed the Collaboration for Environmental Evidence Guidelines and Standards for Evidence Synthesis [27].

### Deviations from the protocol

In this section, we set out any deviations from the protocol and the reasons why these deviations occurred. Due to time and resources, we did not iteratively update the web-based searches nor did we manually search the bibliographies of included studies from the organisational website searches. Additionally, we combined the title and abstract screenings as, in practice, the title/abstract screening took place simultaneously; where an article could be excluded based on the title only, then the abstract was not read. The kappa statistic was used as an initial guide to identify potential weaknesses with elements of the search string and/or its interpretation by reviewers. There was fair agreement on the first consistency check (Cohen’s kappa: 0.38). The disagreements resulted from issues of interpreting and understanding hydrological terminology, particularly where there were slightly different wordings of natural flood management between different countries. Two in-person meetings discussed the examples where there was disagreement over the interpretation. This discussion was used to inform alterations to the screening strategy. Reviewer 1 undertook additional reading on the topic to enhance their understanding of instances where hydrological terminology might be relevant to the topic area. It was recognised that this would not address potential reviewer disagreements in their entirety but given resource issues was deemed a viable step in improving consistency.

At full text screening stage, three reviewers completed the full-text screening and met twice to discuss the consistency checks (discussing 20 out of 677 [2.95%] which was repeated again on 10 out of 677 [1.48%]) and where there was some uncertainty over the status of the article. The codes were checked for consistency based upon 12 articles (4.1%) rather than the 5% of articles suggested in the protocol. This decision was taken owing to the volume of articles versus the available time and resources. Rather than use a member of the team external to the coding strategy to consult on tricky codes, the five reviewers on a weekly basis to discuss any studies that were difficult to code. Using this method ensured consistency over coding as a collective rather than bringing in another reviewer.

In addition, we added more exclusionary criteria around academic standards at full-text stage to exclude Masters theses and blog posts. A total of 78 studies were further excluded at data coding stage. This was partly due to including a larger number of reviewers and careful checking of the included sources. At title, abstract and full text screening stage, the reviewers let articles proceed where there were small doubts over a study’s suitability. In the weekly meetings described above, five reviewers took a conclusive view on the articles which led to their exclusion at data coding stage. The number of codes proposed in the protocol coding strategy were reduced due to time and resources. We recognise that there are consequences placed on the study by these decisions, which we discuss in the section on ‘limitations’.

### Searching for articles

#### Search string

The final search string, formatted for use with Web of Science, adopted for the databases (excepting Google Scholar) is provided in Additional file 3.

We also aimed to be inclusive with the search string and included terms such as ‘sustainable drainage system’ since the definitions of various terms, such as sustainable drainage system, nature-based solutions, and green infrastructure, are porous and may overlap with NFM [26]. However, these sources were either included or excluded at article screening stage depending on the narrow definition of NFM measures in Table 1 at title and abstract screening stage.

All search results were imported into EPPI-reviewer which is a web-based programme that can assist with systematic literature reviews. EPPI-reviewer automatically identified duplicates although some duplicates were later picked up manually at title and abstract screening stage, and also data coding stage. All searches were performed in English and included only articles in written English.

#### Search sources

The search for articles occurred between June and September 2019. We searched bibliographic databases (Table 3), Google Scholar as a web-based search engine, and recognised organisational websites (Table 4) to unearth both academic and grey literature. To search the bibliographic databases, we used the phrase ‘natural flood management’ with Boolean operators (Additional file 3). The Google Scholar search strategy followed the guidance outlined in Haddaway et al. [28] (Additional file 3). We included the first 1000 results from each of the Google Scholar searches. Full-text files were downloaded utilising a University of Manchester Library subscription. All sources were available online using this source so there was no need to access hard copies.

**Table 3:** List of bibliographic databases that were searched for evidence along with the platform and subscription

Database	URL
Academic search premier	https://www.ebsco.com/products/research-databases/academic-search-premier
CAB abstract	https://www.cabdirect.org/
DART-Europe E-theses Portal	http://www.dart-europe.eu/basic-search.php
DOAJ^a^	https://doaj.org/
EThOS^b^	https://ethos.bl.uk/Home.do;jsessionid=4F5C4D13E2BC74ADEB6AD52745760852
EBSCO Host^c^	https://search.ebscohost.com/Login.aspx
GreenFILE	https://www.ebsco.com/products/research-databases/greenfile
International Bibliography of the Social Sciences (IBSS), Sociological Abstracts, and Worldwide Political Science Abstracts	https://search.proquest.com/ibss
ProQuest dissertations and theses^d^	https://search.proquest.com/pqdtglobal
Research fish	https://www.researchfish.net/
Scopus	https://www.scopus.com/standard/marketing.uri
Social science premium collection	https://search.proquest.com/socialsciencepremium?_ga=2.15911286.110795125.1579537448-936894817.1579537448
Web of Science Core Collections • Science Citation Index (1900–present) • Social Science Citation Index (1956–present) • Arts and Humanities Citation Index (1975–present) • Emerging Sources Citation Index (2005–present) • Conference Proceedings Citation Index–Science (1990–present) • Conference Proceedings Citation Index-Social science and humanities (1990––present)	http://login.webofknowledge.com/error/Error?Error=IPError&PathInfo=%2FWOS&RouterURL=http%3A%2F%2Fwww.webofknowledge.com%2F&Domain=.webofknowledge.com&Src=IP&Alias=WOK5

^a^ http://bit.ly/2GFobAB—shortened search string (flood* AND “natural flood management”)

^b^https://ethos.bl.uk/SearchResults.do

^c^ http://bit.ly/2OCRmZ9

^d^Unwieldy number of search results (12,406) in unrelated topic areas (e.g. prehistoric eskimo culture)

**Table 4:** List of organisational websites that were searched for organisational grey literature

Organisation	URL
Catchment Based Approach	https://catchmentbasedapproach.org/
Chartered Institute of Ecology and Environmental Management (CIEEM)	https://cieem.net/
Chartered Institution of Water and Environmental Management (CIWEM)	https://www.ciwem.org/
Climate Adapt	https://climate-adapt.eea.europa.eu/
Community Research and Development Information Service (CORDIS)	https://cordis.europa.eu/
Construction Industry Research and Information Association (CIRIA)	https://www.ciria.org/
Department for Environment Food and Rural Affairs (DEFRA)	https://www.gov.uk/government/organisations/department-for-environment-food-rural-affairs
Environment Agency (EA)	https://www.gov.uk/government/organisations/environment-agency
European Environment Agency	https://www.eea.europa.eu/
Forest Research	https://www.forestresearch.gov.uk/
Institute of Environmental Management and Assessment (IEMA)	https://www.iema.net/
Intergovernmental Panel on Climate Change (IPCC)	https://www.ipcc.ch/
Natural Resources Wales	https://naturalresources.wales/?lang=en
Northern Ireland Environment Agency (NIEA)	https://www.daera-ni.gov.uk/northern-ireland-environment-agency
The River Restoration Centre	https://www.therrc.co.uk/
The Rivers Trust	https://www.theriverstrust.org/
Scottish Environmental Protection Agency (SEPA)	https://www.sepa.org.uk/
United Nations Environment Programme (UNEP)	https://www.unenvironment.org/
United Nations Framework Convention on Climate Change (UNFCCC)	https://unfccc.int/
United Nations International Strategy for Disaster Reduction (UNISDR)	https://www.unisdr.org/
The Woodland Trust	https://woodlandtrust.org.uk

Table 4 details the organisational websites that were searched. For each organisational website, the search terms used in the Google Scholar searches were applied and the first 100 search results from each site were examined and screened for inclusion in line with the study’s eligibility criteria. Only freely available articles and reports were recorded.

#### Estimating the comprehensiveness of the search

Our test list (Additional file 4) was developed collaboratively with an academic advisory group consisting of environmental and social scientists in order to ensure sources covered a range of disciplinary backgrounds. Owing to resource issues, the search string was tested on Web of Science and Scopus only. We aimed for a 100% return rate [18]. In total, 17 of the 18 sources were found. The source that was not found nevertheless contained material outlined in another returned source.

#### Search update

No search update was undertaken.

### Article screening and study eligibility criteria

#### Screening process

Screening took place sequentially at two levels: title/abstract and full text, both screening levels were completed through EPPI reviewer.

#### Eligibility criteria

We followed the ROSES Flow reporting form (see Additional file 2). Eligibility and exclusion decisions were applied at each level. Sources passed onto the next level if there was no explicit indication of the exclusion criteria and also where there was some uncertainty around whether the source could be excluded.

Eligible population: Study addresses current and/or future flood risk,

Is relevant to the UK in terms climate and regulatory context, Identifies the broadly defined biophysical, social, and/or political conditions within which flood risk arises.

Eligible intervention: Study analyses an aspect of the effectiveness of specific, single NFM measures, NFM measures studied have been knowingly and deliberately implemented to manage flood risk.

Eligible comparator: No comparator.

Eligible outcome: Evaluation of the impact of NFM measure on current and/or future flood risk, Impact of biophysical, social, and/or political conditions on NFM and vice versa.

Criteria applied at title and abstract stage:

- Exclude sources which have no applicability to the UK by way of clear differences in climate and regulatory context, i.e. in the same Koppen climate classification and located within the European Union, as discussed in [20].
- Exclude sources which do not cover a specific NFM measure (or measures) listed in Table 1.

Criteria applied at full-text stage:

- Exclude sources which are historical in their evaluation of NFM (e.g. addresses historic inundation levels using paleoecological techniques).
- Exclude sources which amalgamate the impacts of several NFM measures (e.g. at a catchment scale) into an overall assessment of flood risk and therefore do not provide an evaluation of single NFM measures.
- Exclude sources which re-state the benefits of NFM with no original research.
- Exclude sources which do not knowingly and deliberately recognise flood risk as a driver for the NFM measure.
- Exclude sources that are Masters theses and blog posts based on academic standard.

The final exclusionary criteria at full-text stage were added to after the protocol was developed as it became clear that some papers may describe NFM measures but did not have current or future flood risk as a driver. A criterion was included at full-text stage to reject Masters theses and also blog posts because it was difficult to judge the academic standard.

EPPI-reviewer was used for title/abstract and full-text screening. To ensure that the review process was consistent, a consistency check was performed on a random selection of articles (N: 1638, 8.5%, randomised by EPPI-reviewer). There was fair agreement on the first consistency check (Cohen’s kappa: 0.38). Time and resources precluded us from repeating the consistency check until the Cohen kappa reached 0.60. We decided to take a wide view on terminology and include if in doubt for the next screening stage. In addition, we intended the title and abstract screening to be undertaken by a single reviewer. However, owing to the volume of articles (see results), two reviewers split the task. Reasons for exclusion at title/abstract screening stage were based on population, mainly where a paper was not within the correct Koppen climate classification. All duplicates were removed through both automated removal and manual screening in EPPI reviewer. At full text screening stage, 20 out of 677 [2.95%] were included in the consistency check with three reviewers. There was disagreement on 4 out of 20 articles as a result of including an extra reviewer. The review was repeated one more time with 10 out of 677 articles [1.48%] where there was complete agreement.

None of the identified sources were authored by a member of the review team.

#### Study validity assessment

Given the available resources and the diversity of the articles included in the systematic map, we did not assess individual articles for quality such as considering their validity and reliability.

#### Data coding strategy

Each included article was coded in EPPI-reviewer using a standardised set of codes (Additional file 1). At this stage, a further two reviewers were included making a total of five reviewers at full text coding stage. The codes were checked for consistency based upon 12 articles (4.1%). There was some disagreement over the application of codes, particularly around scale and so the choices for this were simplified from being area based to being a simple yes–no between catchment scale or not, with further codes to capture those studies that examined issues at more than one scale; ‘cross-scale’ studies examined the interactions between scales, and ‘multiple scales’ indicated studies that looked at multiple scales without necessarily considering the interaction between them.

More detailed notes were also added to the codes in EPPI-reviewer to ensure consistency across reviewers. During the coding process, the five reviewers were in contact on a weekly basis to discuss any articles where coding was difficult to come to a collective agreement on how best to code these articles. By using this method, there were no disagreements that could not be resolved and so it was not necessary to use another member of the team, a strategy that had been used in previous reviews and as described in the protocol [e.g. 29]. It was also not felt necessary to contact any authors for further information or to consult the advisory group. Given that sources at previous screening stages were included even if in any doubt, further discussions between a larger number of reviewers led to articles being excluded at coding stage also.

The following categories of data were extracted from each article:

- Unique article ID information.
- Bibliographic information (including publication type)
- Information on NFM measure implemented.
- Information on types of effectiveness studied.
- Information on type of flooding addressed and scale at which the NFM intervention was aimed.
- Information on the scientific basis of the study and the type of data collected
- Information on the forms of NFM effectiveness assessed.
- Information on the characterisation of climate change.

NFM measures were coded following the definition on NFM from the Environment Agency and outlined in Table 1 [1]. Types of effectiveness followed Pope et al. [22] and are outlined in the systematic map protocol [20]. For these criteria, each article was coded in the systematic map as reporting:

1. Substantive effectiveness
2. Procedural effectiveness
3. Transactive effectiveness
4. Normative effectiveness (in terms of climate change mitigation and adaptation)
5. No form of effectiveness examined

Where an article assessed more than one form of effectiveness, the requisite number of codes was selected.

Normative effectiveness, particularly given NFM’s recognised ability to contribute to multiple societal goals, was narrowed down to focus only on the contribution of NFM to climate change goals as we wanted to keep the coding manageable and wanted to examine the consideration of climate change. The original protocol suggested that climate change would be coded as part of normative effectiveness. However, in practice, we decided to include a separate code to discern the nature of how a particular source engaged with climate change. Where studies mentioned climate change as a potential motivation or rationale for the study, or where a study made claims that NFM could contribute to climate change mitigation and adaptation but contained no further data such as projections and scenarios, this was considered to be a ‘rationale’ for the study. Only studies that worked with modelled data on climate change (e.g. climate change projections) and/or explicitly asked qualitative questions around climate change were deemed to have addressed climate change with a ‘substantive focus’. Studies that did not mention climate change were coded as ‘no consideration’.

We dispensed with time scale of the study as a code as it did not pertain to all the types of effectiveness and so was not a useful variable. Additionally, we originally planned to include ‘rural’ and ‘urban’ in the coding. However, owing to time and resources, we decided that these categories were less pertinent to the focus on types of effectiveness.

#### Data mapping method

Extracted data were exported from EPPI-reviewer and analysed in Excel. The frequency of general characteristics of the studies (e.g. geographic location, year of publication etc.) were examined in tables, histograms and heatmaps.

Cross-tabulations were produced for certain variables that were relevant to the systematic map objectives such as type of effectiveness versus NFM measure. Heat maps were produced in Excel to identify where there were clusters of forms of effectiveness with certain NFM measures. The data mapping allowed for the identification of which forms of effectiveness are considered in NFM studies, and relates to the disciplinary foci of studies. The recommendations for policy and research were derived from these analyses.

## Review findings

### Review descriptive statistics

**Fig. 3:**
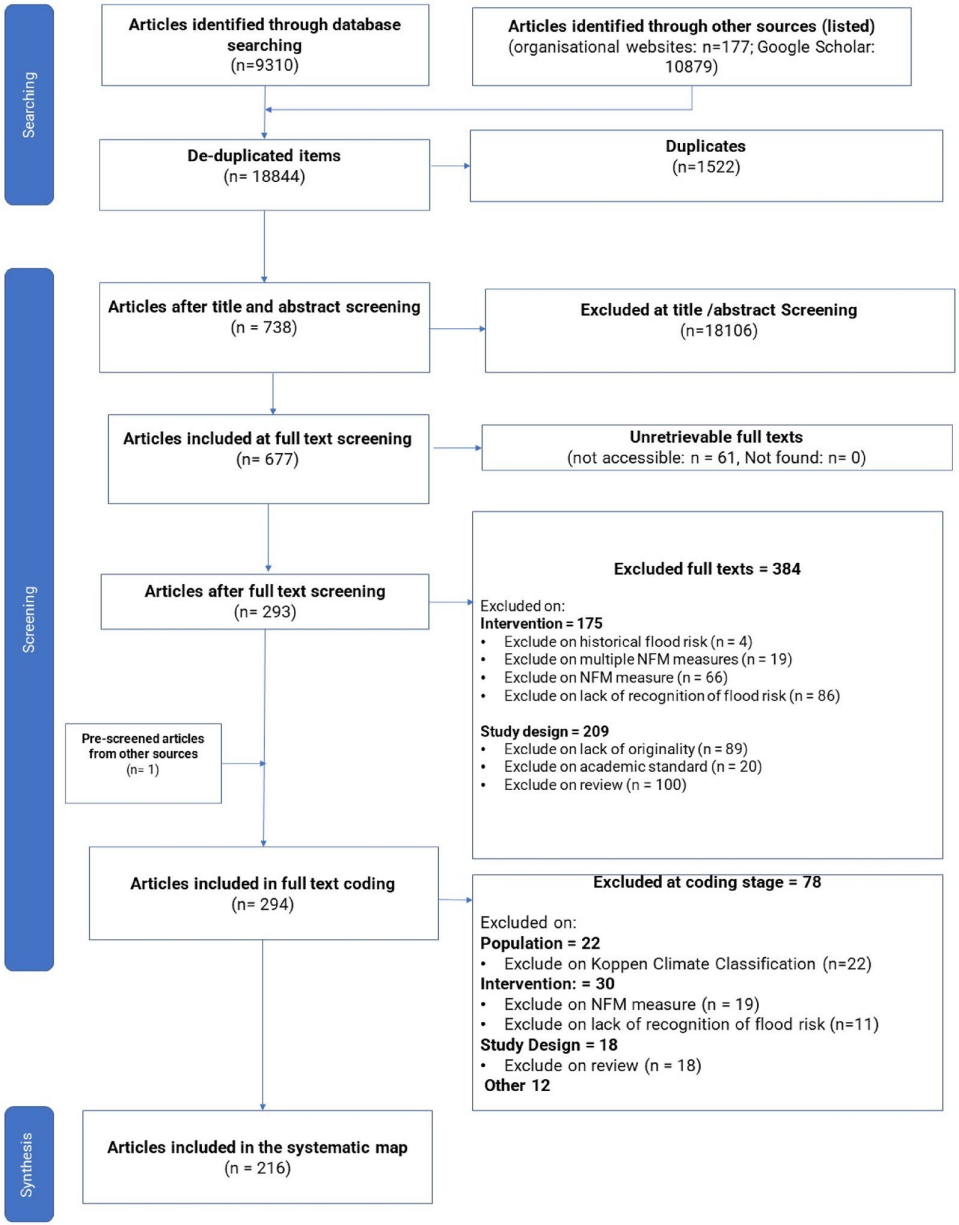
An overview of the systematic mapping process following ROSES guidance [26]

The search took place between June 2019 and September 2019. In total, 20,130 results were retrieved (Fig. 3). Searches of organisational websites identified in Table 4 yielded 177 relevant articles. 1522 duplicates were identified automatically by EPPI reviewer or during the title and abstract screening stage. Most articles were excluded at the title and abstract screening stage due to irrelevance to NFM (N = 9483), for not including an NFM measure stated in Table 1 (N = 739), or for not being in the identified climatic region (N = 3334) (Fig. 3). Review articles were permitted to go through to full text screening stage in case any new data was given within the article.

At full text screening stage, a further 384 articles were excluded. This was based on the intervention being inappropriate (N: 175) due to results that were based on historical flood risk (N: 4), considered multiple NFM Measures (N: 19), excluded on the NFM measure (N: 66) or because of the lack of recognition of flood risk (N: 86). The latter criteria typically occurred where a source considered wider ecosystem-based adaptation rather than flood risk per se. 61 articles were irretrievable as a full text version. Study design (N: 209) resulted in further exclusions based on lack of originality (where a study did not present new data) (N: 89), academic standard (to exclude Masters theses and non-peer-reviewed blog posts) (N: 20), and review articles (N: 100). Originality was intended to capture those sources where data was reused over multiple articles versus review articles which synthesise data.

294 texts went forward for data coding. At this stage, a further 78 sources were excluded due to: climatic region (n: 22), a lack of original data (n: 18), non-inclusion of identified NFM measures (n: 19), or for a lack of recognition of flood risk (n: 12), ‘Other’ was chosen for 10 sources. Three sources had more than one reason for exclusion. These excluded sources were due to our collective checking on criteria and discussing the articles to be coded. At earlier stages in the process, articles proceeded if there was any doubt over their suitability. A continual process of iteration and conversation over the interpretation of the criteria, brought about by having more reviewers at later stages, led to refinement. For example, there was some disagreement on whether data from case studies that sat on the boundary of a climate zone were to be included and, in the end, it was decided to exclude these.

In total, 216 single study articles were included in the final systematic map. Output from EPPI-reviewer of excluded articles at full text screening stage with exclusion criteria as well as exclusions at coding stage are listed in Additional File 5. The articles that were coded, and the coding data, can be found in the systematic map database in Addition File 6. A ROSES reporting form is included in Additional File 2.

#### Temporal spread

**Fig. 4:**
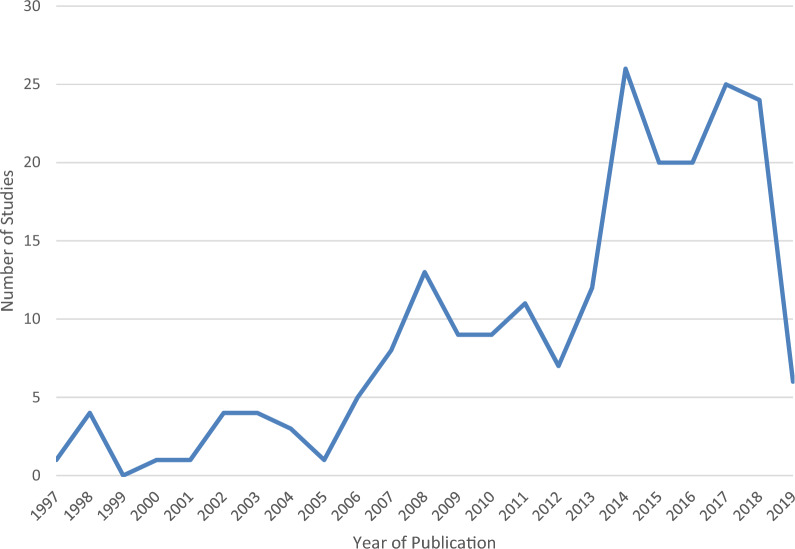
Number of studies by year of publication

The earliest study included in the systematic map was in 1997. Generally, the number of studies increased overall up to the present day, with the largest proportion occurring after 2010 which indicates growing interest in NFM as a method of reducing flood risk (Fig. 4). In terms of effectiveness, no discernible trends could be identified dependent on the year of study. Consideration of climate change increases marginally in later work (2010–2019; 36.0%) than earlier work (1997–2009; 30.3%). There is a drop off for 2019 because the search took place in the middle part of that year and therefore excludes articles published after September 2019.

#### Geographical spread

**Fig. 5:**
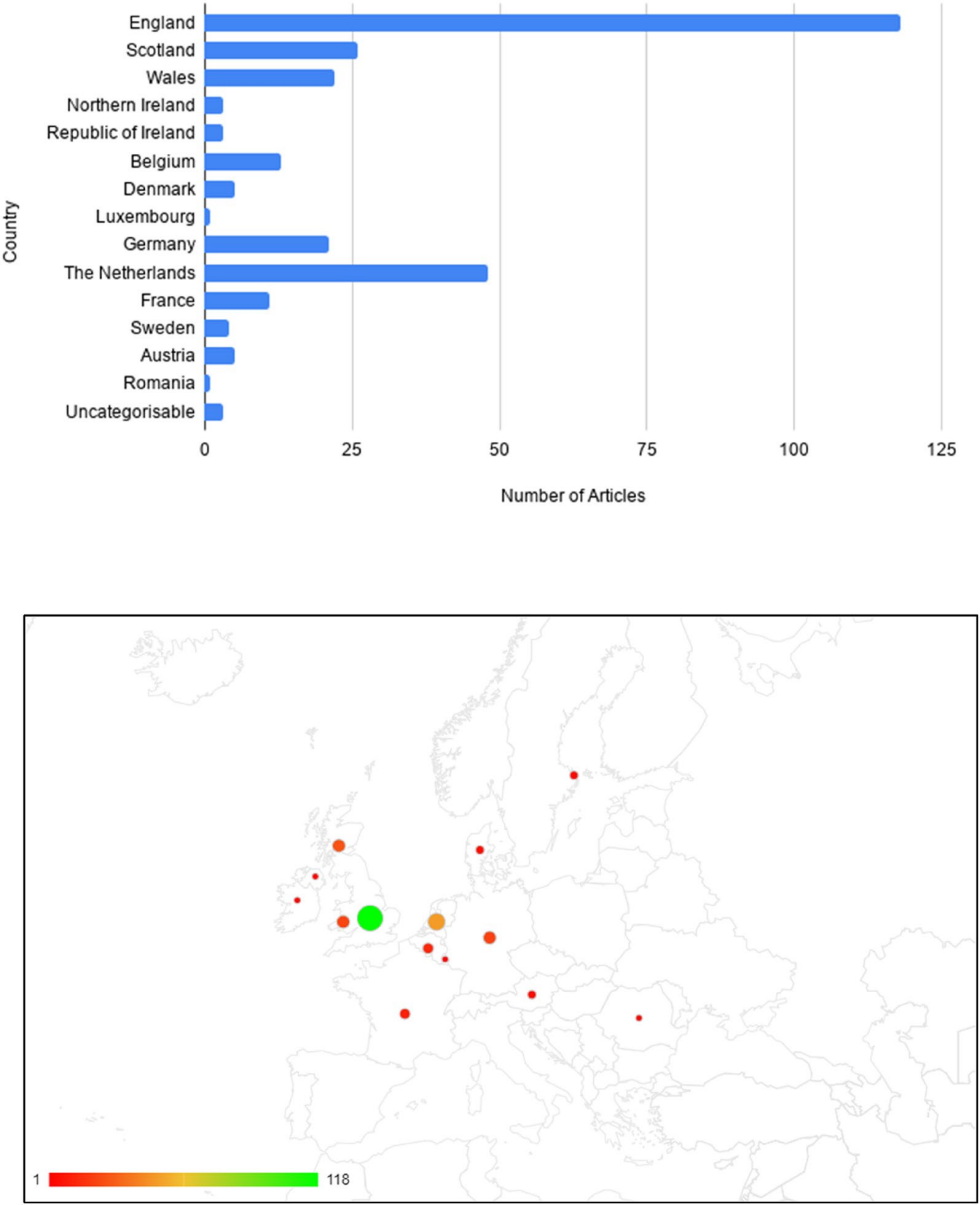
Geographic Heatmap of the distribution of articles

The overwhelming number of studies were located in England (n: 118) and followed by the Netherlands (N: 48), Scotland (N: 26), Wales (n: 22) and Germany (N: 21). This means that much of the UK-relevant studies are produced in the UK, and there is limited research from other countries with the exception of the Netherlands. However, this could be biased due to the systematic map only considering studies in written English (Fig. 5).

The proportion of studies by type of NFM varied across different countries (Additional file 8). England dominated studies in headwater drainage (N: 10: 90.9%), managed realignment (N: 34, 59.6%), and leaky barriers (N: 12, 80%). The Netherlands had a significant proportion of research in beach nourishment (N: 19, 51.4%) and sand dunes (N: 5, 22.7%). Cross-slope, Floodplain and Riparian woodlands and offline storage are much more wide-spread across different countries.

#### Type of flooding

**Fig. 6:**
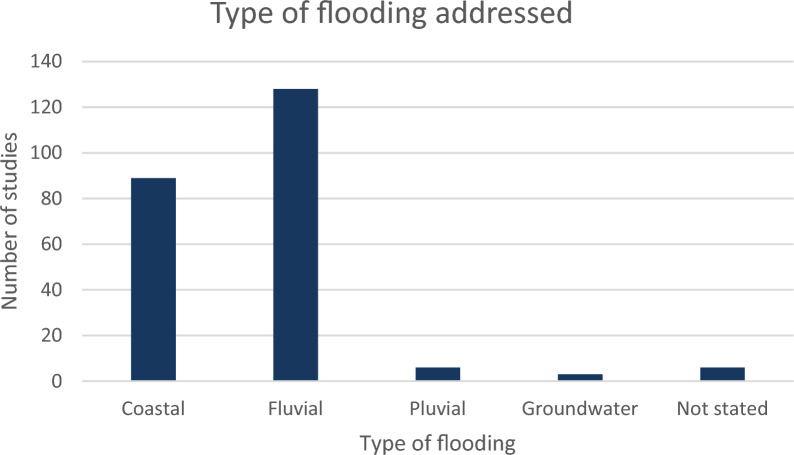
Type of flooding addressed

Sources were dominated by a consideration of coastal (N: 89; 41.2%) and fluvial flooding (N: 128, 59.3%). The lower amounts of sources related to pluvial flooding, which is generally more prevalent in urban areas, and may be due to the exclusion of SuDS as an NFM measure in the definition cited above and in Table 1 (Fig. 6).

#### Scale

Data coding captured the scale at which studies took place. Where identified, the majority (N = 137, 63.4%) did not take place at catchment scale. A total of 42 sources examined catchment scale (19.4%) whilst 6 sources (2.7%) examined cross-scale interaction of the effectiveness of a given NFM measure (i.e. considered how different scales worked in terms of a given NFM measure’s identified form of effectiveness). A further 16 (7.4%) examined evidence at multiple scales without necessarily considering how those scales interacted. In 17 sources, scale was not applicable or identifiable. Two sources were double coded as these ostensibly examined issues at catchment scale but also included some discussion of cross-scale interactions. These findings are in line with other reviews that indicate that catchment scale research is difficult to evaluate with NFM even though it is thought that effectiveness measured at catchment scale will be able to uncover the multiple benefits of NFM [e.g. 4]. Given the highly localised scales of NFM implementation and appraisal in a UK context, it was deemed inappropriate to the aims of the study to include studies which evaluated the aggregated impact of multiple NFM measures.

### Mapping the quantity of studies relevant to the question

This systematic map principally sought to understand the different forms of effectiveness that sources evaluate in relation to NFM with a supplementary question around whether sources engaged with climate change and future flood risk. This section sets out the results of analysis in relation to the forms of NFM effectiveness.

Overall, only 20 studies did not consider any form effectiveness, meaning that 90.7% of included studies considered some type of effectiveness (Table 5; See Additional file 6 for the coded data). Studies were more likely to assess substantive effectiveness (N: 135, 62.5%) followed by procedural effectiveness (N: 91, 42.1%). This may indicate that debates are predominantly framed in terms of ‘Does NFM work from the perspective of reducing flood risk?’ and ‘what are the correct processes for implementing NFM?’.

**Table 5:** Descriptive statistics on NFM effectiveness

NFM effectiveness type	Number of studies
Procedural	91
Substantive	135
Transactive	55
Normative	40
None of the codes above	20

Note that some studies were double coded

The highest proportion of studies considered only one type of effectiveness (N: 110, 50.9%) (Table 6). Where studies combined effectiveness, these were most likely to only include two types of effectiveness (N: 53, 24.5%) which were most commonly substantive and procedural effectiveness (N: 17, 34.7%). Only 7 (3.24%) studies combined all types of effectiveness.

**Table 6:** NFM effectiveness and the number of studies considering more than one type of effectiveness

Effectiveness combinations	Number of studies per combination	Total number of studies
None	20	20
Procedural	39	
Substantive	64	
Transactive	7	
Normative	0	110
Procedural-Substantive	17	
Procedural-transactive	11	
Procedural-normative	0	
Substantive-transactive	8	
Substantive-normative	13	
Transactive-normative	3	52
Procedural-substantive-transactive	8	
Procedural-substantive-normative	7	
Procedural-transactive-normative	2	
Substantive-transactive-normative	10	27
Procedural-substantive-transactive-normative	7	7
Total number of studies		216

Additional file 7 presents a heat map showing how forms of effectiveness are distributed across different NFM measures. Substantive effectiveness dominates in soil and land management (N: 16, 69.6%) and floodplain and floodplain wetland restoration (N: 34, 37.8%). River restoration (N: 25, 37.3%) and managed realignment (N: 14, 33.3%) more commonly consider procedural effectiveness. This may be due to the significant impacts on local communities that river restoration and managed realignment schemes may have; therefore, there is more attention paid to due process around stakeholder and community involvement. Normative effectiveness, here defined as the extent to which an NFM measure helps to realise climate change mitigation and adaptation aims, is low across all measures but proportionately higher when considering floodplain and floodplain wetland restoration (N: 12, 13.3%) and saltmarsh and mudflats (N: 10, 20.4%). Transactive effectiveness is proportionately higher when considering river restoration (N: 16, 23.9%) and floodplain and floodplain wetland restoration (N: 18, 20%).

#### Effectiveness—flood risk and scale

Different effectiveness assessments are spread across coastal and fluvial flooding in similar trends with substantive effectiveness accounting for the highest proportions, followed by procedural effectiveness then transactive effectiveness. Normative effectiveness is the least considered in both flood types (See Table 7).

**Table 7:** Type of effectiveness by type of flooding

Type of flooding	Procedural	Substantive	Transactive	Normative
Coastal	38	49	18	15
Fluvial	53	80	35	23
Groundwater	1	2	1	1
Pluvial	2	5	2	4
Not stated	4	3	3	2

Note that some sources were double-coded

**Fig. 7:**
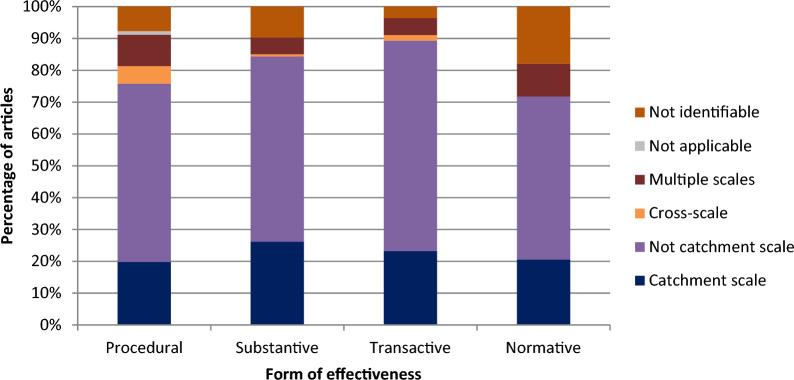
Percentage of articles examining type of effectiveness according to scale of study. Note that some studies considered more than one form of effectiveness

Figure 7 shows the type of effectiveness examined dependent on the scale at which the study took place. Sources examining NFM measures at catchment scale were more likely to consider substantive effectiveness (N: 35, 47.3%) and least likely to consider normative effectiveness (N: 8, 10.8%). Sources examining NFM measures that were not at catchment scale had similar proportions to catchment scale studies although were marginally more likely to consider procedural effectiveness (56% vs 19.8%). The highest percentage of cross-scale studies contained examinations of procedural effectiveness (N: 5, 71.4%). Studies occurring at multiple scales were marginally more likely to consider procedural effectiveness (N: 9, 31.9%) followed by substantive effectiveness (N: 7, 30.4%). This may be due to flood risk management occurring at multiple scales and therefore any account of due process would need to take account of these multiple scales.

#### Consideration of climate change

As a sub-question, the systematic map looked at the quantity of studies that considered climate change. Just under 75% of studies mention climate change in some form either giving the topic systematic consideration (24.5%) or where climate change was cited in the rationale for the study (49.1%). Whilst almost half of papers acknowledge climate change as being one of the spurs for the study, this means that only around one-quarter include a systematic consideration of the potential impact of climate change through, for example, utilising climate projections within modelling NFM effectiveness or addressing the fundamentals of climate adaptation planning.

Table 8 shows the percentage of papers that do not consider climate change, cite climate change as a rationale for the study, or contain substantive consideration of climate change. Less than one-fifth of the total papers for run-off pathway management, catchment woodlands, soil and land management, leaky barriers and floodplain and floodplain restoration consider climate change substantively. This suggests that there is a gap in knowledge around how climate change may impact upon the effectiveness of a given measure. Around half of papers considering sand dunes and cross-slope woodlands provided substantive consideration of climate change. The highest percentage of ‘no’ consideration of climate change came for run-off pathway management and headwater drainage although the latter only a small sample of papers (N: 11).

**Table 8:** Percentage of papers for each measure and their consideration of climate change

NFM measure	% of papers vs consideration of climate change		
	No	Rationale	Substantive
Run-off pathway management	61.5	30.8	7.7
Catchment woodlands	35.7	50	14.3
Soil and land management	45.7	40	14.3
Leaky barriers	38.5	46.2	15.3
Floodplain and floodplain Restoration	44.3	36	19.7
Headwater drainage	60	20	20
River restoration	47.7	31.8	20.5
Managed realignment	7.4	65	27.5
Offline storage area	35.7	35.7	28.6
Floodplain woodlands	29.4	41.2	29.4
Saltmarshes and mudflats	9.1	57.6	33.3
Beach nourishment	24.1	41.3	34.5
Riparian woodlands	45	20	35
Other/uncategorisable	45.4	18.2	36.4
Cross-slope woodlands	33.3	22.2	44.4
Sand dunes	13.3	33.3	53.3

#### Focus of design

The studies varied in terms of research design according to the type of effectiveness being measured. Substantive effectiveness was most likely to be assessed via quantitative experimental designs. Procedural effectiveness was more likely to be assessed via qualitative social scientific research (e.g. interviews and focus groups). Higher proportions of mixed methods and interdisciplinary research was found where studies assessed transactive and normative research.

**Fig. 8:**
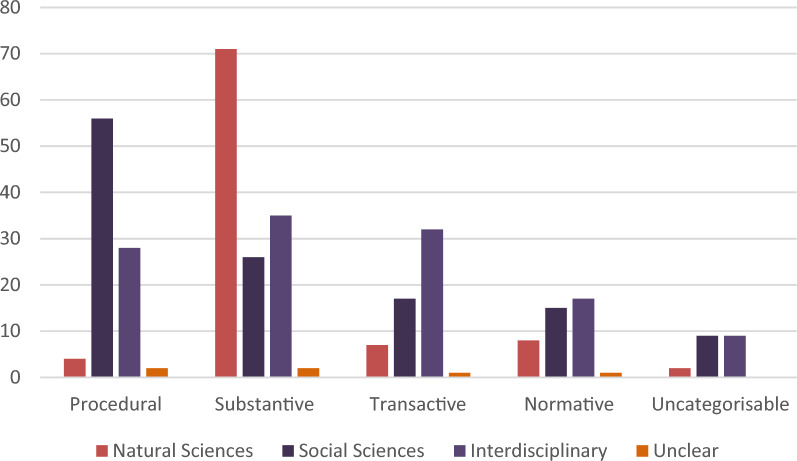
Scientific basis of the study associated with effectiveness

Figure 8 shows the type of effectiveness associated with the scientific basis of the study (natural sciences, social sciences or interdisciplinary). The natural sciences are dominated by considerations of substantive effectiveness (N: 71), although with some consideration with other forms of effectiveness. The social sciences consider a spread of different forms of effectiveness with an emphasis on procedural effectiveness (N: 56). Interdisciplinary studies are also spread across a consideration of different forms of effectiveness with a slight emphasis on substantive effectiveness (N: 35).

**Fig. 9:**
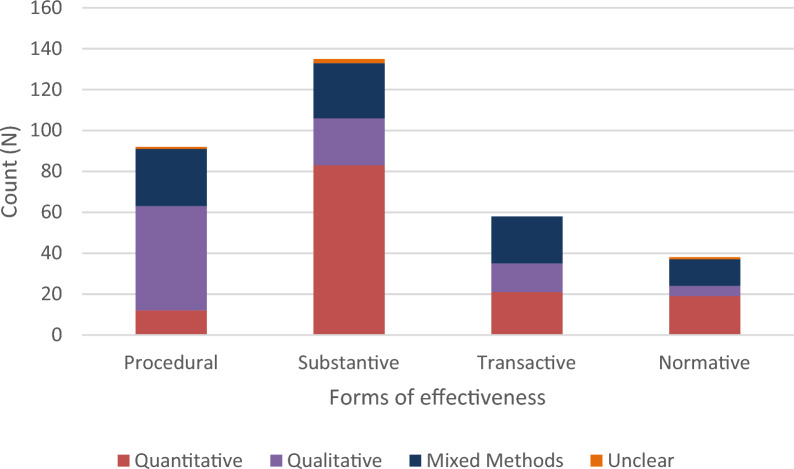
Type of effectiveness by data collection method (note that some sources were double coded)

Figure 9 shows the type of effectiveness associated with particular data collection methods. Qualitative data collection is most commonly associated with sources examining procedural effectiveness (N: 50). Studies that use mixed methods are more common in studies examining transactive (N: 23). Quantitative data collection is least commonly associated with sources examining procedural effectiveness (N: 11). Quantitative data collection is most commonly associated with sources examining substantive effectiveness (N: 81). Quantitative (N: 18) and mixed methods (N: 13) forms of data collection are most likely to consider normative effectiveness.

In order to come to a more holistic assessment of the effectiveness of NFM, then it is clear that more studies need to include more types of effectiveness, particularly in the types of study that tend to focus on substantive effectiveness only where only one NFM outcome is being evaluated.

In terms of their consideration of climate change, Table 9 shows that social science and interdisciplinary studies are proportionately more likely to give climate change systematic consideration.

**Table 9:** Consideration of climate change depending on scientific basis and data collection method

Scientific basis	Data collection methods	Consideration of climate change		
		No consideration (N)	As a rationale (N)	As a substantive element (N)
Natural sciences	Quantitative	35	22	13
	Qualitative	1	0	0
	Mixed methods	1	2	0
Social sciences	Quantitative	2	2	7
	Qualitative	17	27	12
	Mixed methods	4	6	4
Interdisciplinary	Quantitative	3	5	4
	Qualitative	2	5	1
	Mixed methods	8	12	12

This study did not look assess the quality of the available evidence

## Limitations of the systematic map

There are a number of limitations of this systematic map. The search strategy was broad to be inclusive of the articles found and the loose categorisation of the outcome as ‘Impact of biophysical, social, and/or political conditions on NFM and vice versa’ meant that high numbers of irrelevant articles were brought forward. This meant that time and resources were disproportionately spent on excluding articles over doing a more detailed coding exercise. For example, we did not examine the nature of the form of primary data associated with sources (e.g. the use and type of modelling). This meant that there may be wider patterns in the sources included in the systematic map that are not reported here.

There may be some types of NFM that are not included under the adopted EA definition [1]: hedgerows, for example. However, we followed the search string in the protocol that was based on a previously published definition. All classifications are imperfect and, as a result, some NFM measures may have been missed.

A decision was taken to focus only on single NFM measures. However, NFM measures are often used in conjunction with one another and so there is a gap in the systematic map of the consideration of effectiveness where NFM measures are used as a system to reduce flood risk. In total, 19 studies that examined NFM systematically as a combination of measures were excluded. Examining NFM at small scales means that their multiple benefits at catchment scale are not captured. With more resources, effectiveness within NFM systems should be built into future reviews.

The conceptualisation of effectiveness, from a policy point of view, could be reworked in any future reviews. This study defines effectiveness as the extent to which NFM measures help to realise goals. However, policy goals often change. If goals do not remain static, then measurements of effectiveness are being made against shifting baselines. Furthermore, with more resources, the coding of effectiveness types could be further developed to assess the extent to which the attributes associated with effectiveness have been captured. Each form of effectiveness could potentially be comprised of multiple components and this study took a basic consideration of the categories of effectiveness owing to time and resources.

A further limitation occurs in our coding of normative effectiveness to examine the climate change issue only; normative effectiveness relates to broader ideals which, with NFM, can include increased biodiversity, health and well-being, contribution to placemaking, and so on. In order to keep the coding at a manageable level due to time and resources and to address the systematic map sub-question around climate change, we coded only for climate change which means that the map could have gone further in terms of understanding effectiveness from a greater range of the broader ideals that can be realised with NFM. Moreover, the coding of climate change is relatively simplistic by only coding in terms of whether climate change was considered as a substantive component of the study, as a rationale only, or where there was no consideration. The codes could be developed further, particularly within the substantive component, to consider the way in which articles use data on climate change.

There are two related consequences of excluding masters theses and blog posts. Firstly, that original and relevant evidence could have been excluded. At the full text stage this amounted to 20 sources. These are, in principle, part of existing evidence on the topic. Secondly, that in excluding these, the study’s utility as a contribution is undermined as it misses part of the evidence based. To address these weaknesses, however, would require a quality check. This, we suggest, should be a task for any subsequent systematic review.

There are limitations in terms of the screening and coding process. A larger than average number of reviewers and coders meant that there was constant iteration and discussion over the interpretation of the codes. It is therefore possible that there is some inconsistency in the screening process. In this case, we recommend having a higher percentage of articles pre-screened to account of disagreements where more than one or two reviewers are used.

Additionally, we were not able to find one source on our test list. This raises the possibility that relevant sources have been missed in the search strategy which we acknowledge. We reviewed articles only in English, therefore there may be the risk of bias towards studies located within the UK in terms of the geographic distribution of sources.

## Conclusion

This systematic map set out to discover how UK-relevant NFM studies consider the effectiveness of these measures. A sub-question included the way that climate change was considered in these studies, if at all. This section considers the implications for policy/management and research.

## Implication for policy/management

In terms of effectiveness, this systematic map established that that the consideration of effectiveness within NFM research is predominantly based around substantive effectiveness and procedural effectiveness. Transactive and normative forms of effectiveness are less likely to be evaluated. It appears, therefore, that the evaluation of NFM effectiveness is driven by the type of evidence-based approaches to flood risk management that currently dominate decision making and resource allocation in the UK. Lane [30] notes that flood risk management is rooted in a quantitative determination of economic losses, which helps to explain why substantive interpretations of NFM effectiveness are dominating NFM research (and therefore research funding) as evaluations of the impact of measures on runoff volumes and river levels can support calculations on related implications on flood extent and damage.

It is encouraging to find that research studies are evaluating NFM effectiveness from a substantive perspective, where the impact of measures on reducing flood risk is the key focus. This systematic map has also demonstrated that some NFM studies are also focusing more broadly on NFM effectiveness and what constitutes and effective NFM measure.

Policy makers should take note of the finding of this systematic map that proportionally fewer studies systematically examine the implications and dynamics of climate change for NFM, although they may make claims with regards to the benefits of NFM to future flood risk. Given the potential durability of NFM measures, it is not only important to consider their potential benefits to reducing flood risk under various climate scenarios, but also to understand the extent to which NFM is at risk from climate change (e.g. under drought conditions). This will help to increase confidence around the contribution of NFM to climate change mitigation and adaptation, and may also encourage consideration of approaches to increase the resilience of NFM measures potentially impacted by changing climate parameters and extreme weather events.

## Implication for research

This systematic map tried to understand how effectiveness was assessed in NFM research. The systematic map shows that there is a need for robust research that substantively considers NFM and their contribution to climate change adaptation and mitigation at various scales and for different measures.

Although a broadening of the conceptualisation of NFM effectiveness would be valuable, this research finds an existing situation where different forms of NFM effectiveness are not being evaluated to the same extent as substantive effectiveness. NFM studies appear to be paying less attention to themes including the wider benefits of NFM (their normative effectiveness), such as their contribution to climate change mitigation and adaptation goals. It can be argued that such themes should be considered more commonly, particularly as the issues with evaluating substantive forms of effectiveness are increasingly being recognised. For example, Dadson et al. [10] note that NFM is less effective, in terms of reducing water flows, during extreme rainfall events. This is not a reason to therefore limit NFM implementation, but such findings do emphasise that NFM should only ever be part of a wider suite of NFM strategies and crucially also highlight the value of considering other forms of effectiveness when evaluating NFM measures. Indeed, NFM measures may be effective for reasons including supporting the achievement of climate change mitigation goals, and these other forms of effectiveness should be placed on a more equal footing with substantive interpretations to enable other aspects of NFM measures to be recognised and accounted for in decision making. Although there is value in evaluating NFM from a broader perspective it is likely that challenges will nevertheless be faced, such as those faced when considering the substantive effectiveness of NFM.

This study also found that a significant proportion of the coded sources were based on social science methods, particularly examining procedural effectiveness, which contradicts a perception that the evidence base is largely dominated by the natural sciences [3]. There is increasing reporting of interdisciplinary studies and these are more likely to examine more forms of effectiveness. This more holistic approach should be encouraged. There is a continued need for funding to focus on interdisciplinary studies. Such studies are more likely to include different forms of effectiveness in their analysis and will enable a more holistic assessment of NFM measures. This research uncovered many more social science studies compared to previous reviews which suggests some attention being paid to the procedural aspects around NFM and how communities are involved in their implementation. However, the studies can tend to be disparate in their representation in different journals. Therefore, more primary research is needed to consider different forms of effectiveness for any given NFM measure.

This study identified that whilst climate change is often cited as a rationale for a study, climate change is often not systematically considered. More attention needs to be given to how NFM may function under different climate scenarios. Not only does the contribution of NFM to reducing flood risk in a changing climate need to be addressed, but the potential risk that climate change may bring to the effectiveness of NFM measures also needs to be supported by evidence. Indeed, whilst NFM measures and other green infrastructure measures are identified as potential tools to help us adapt to climate change and to become more resilient, it should be remembered that such measures are themselves at risk of climate change [31]. The lack of substantive engagement with future climate change scenarios suggests significant opportunities around understanding the extent to which NFM measures are effective given future climate change projections. This may entail further work around developing approaches to enable UK Climate Change projections to be better used in research.

Future research that follows our model of understanding different forms of effectiveness could usefully widen the codes associated with normative effectiveness to include the understanding of NFM’s contribution to increasing biodiversity, health and well-being and other multiple benefits associated with NFM implementation.

## Supplementary Information

**Additional file 1.** Data coding framework detailing information to be extracted or coded from relevant studies in the final systematic map database.

**Additional file 2.** ROSES for systematic maps form.

**Additional file 3.** Boolean format for search string and Google Scholar Search Strategy.

**Additional file 4.** Search string assessment against test list.

**Additional file 5.** List of articles excluded at full text/coding stage with unretrievable texts.

**Additional file 6.** Full coding report for the systematic map.

**Additional file 7.** Table 9. Heat map showing type of effectiveness versus measure.

**Additional file 8.** Table showing percentage of studies per measure for each included country.

## Data Availability

All data generated and/or analysed during the current study are included in this published article [and its additional files].
