# Supplementary material for: What approaches exist to evaluate the effectiveness of UK-relevant natural flood management measures? A systematic map
Source: Environ Evid. 2023 May 23;12:12. doi: 10.1186/s13750-023-00297-z (PMC11378772; doi:10.1186/s13750-023-00297-z)
Supplement: Supplementary file 3 — Additional file 3. Boolean format for search string and Google Scholar Search Strategy. [file 13750_2023_297_MOESM3_ESM.docx]

# Additional file 3

# Boolean format search string for database searches

Flood*

AND

(“natural flood management" OR "nature based solution*" OR "green infrastructure" OR "building with nature" OR "work* with natural processes" OR “ecosystem-based adapt*” OR “sustainable urban drainage system*” OR “sustainable drainage system*” OR “ecosystem service*” OR “river restoration” OR “floodplain restoration” OR “leaky barriers” OR “offline storage area*” OR “catchment woodland*” OR “floodplain woodland*” OR “riparian woodland*” OR “cross-slope woodland*” OR “soil and land management” OR “headwater drainage” OR “run-off pathway” OR “saltmarsh and mudflat*” OR “sand dune*” OR “beach nourishment” OR “manage* retreat*” OR “manage* realignment” OR “enhanced hillslope storage” OR “runoff attenuation” deculvert* OR “slow the flow”)

# Google Scholar search strategy

**Advanced search**

Search one

With all of the words: flood risk

With the exact phrase: natural flood management

With at least one of the words: adapt resilience

All sources extracted

Search two

With all of the words: flood risk

With the exact phrase: nature-based solution

With at least one of the words: adapt resilience

All sources extracted

Search three (last 2 pages)

With all of the words: flood risk

With the exact phrase: green infrastructure

With at least one of the words: adapt resilience

First 1,000 sources extracted

Search four

With all of the words: flood risk

With the exact phrase: blue infrastructure

With at least one of the words: adapt resilience

All sources extracted

Search five

With all of the words: flood risk

With the exact phrase: building with nature

With at least one of the words: adapt resilience

All sources extracted

Search six

With all of the words: flood risk

With the exact phrase: working with natural processes

With at least one of the words: adapt resilience

All sources extracted

Search seven (last 2 pages)

With all of the words: flood risk

With the exact phrase: ecosystem-based adaptation

With at least one of the words: adapt resilience

First 1,000 sources extracted

Search eight

With all of the words: flood risk

With the exact phrase: sustainable urban drainage system

With at least one of the words: adapt resilience

All sources extracted

Search nine

With all of the words: flood risk

With the exact phrase: sustainable drainage system

With at least one of the words: adapt resilience

All sources extracted

Search ten (last 2 pages)

With all of the words: flood risk

With the exact phrase: ecosystem service

With at least one of the words: adapt resilience

First 1,000 sources extracted

Search eleven

With all of the words: flood risk

With the exact phrase: river restoration

With at least one of the words: adapt resilience

First 1,000 sources extracted

Search twelve (last 2 pages)

With all of the words: flood risk

With the exact phrase: floodplain restoration

With at least one of the words: adapt resilience

First 1,000 sources extracted

Search thirteen

With all of the words: flood risk

With the exact phrase: leaky barriers

With at least one of the words: adapt resilience

All sources extracted

Search fourteen

With all of the words: flood risk

With the exact phrase: offline storage area

With at least one of the words: adapt resilience

All sources extracted

Search fifteen

With all of the words: flood risk

With the exact phrase: catchment woodland

With at least one of the words: adapt resilience

All sources extracted

Search sixteen

With all of the words: flood risk

With the exact phrase: floodplain woodland

With at least one of the words: adapt resilience

All sources extracted

Search seventeen

With all of the words: flood risk

With the exact phrase: riparian woodland

With at least one of the words: adapt resilience

All sources extracted

Search eighteen

With all of the words: flood risk

With the exact phrase: cross-slope woodland

With at least one of the words: adapt resilience

All sources extracted

Search nineteen

With all of the words: flood risk

With the exact phrase: soil and land management

With at least one of the words: adapt resilience

All sources extracted

Search twenty

With all of the words: flood risk

With the exact phrase: headwater drainage

With at least one of the words: adapt resilience

All sources extracted

Search twenty-one

With all of the words: flood risk

With the exact phrase: run-off pathway

With at least one of the words: adapt resilience

All sources extracted

Search twenty-two

With all of the words: flood risk

With the exact phrase: saltmarsh and mudflat

With at least one of the words: adapt resilience

All sources extracted

Search twenty-three

With all of the words: flood risk

With the exact phrase: sand dune

With at least one of the words: adapt resilience

First 1,000 sources extracted

Search twenty-four

With all of the words: flood risk

With the exact phrase: beach nourishment

With at least one of the words: adapt resilience

First 1,000 sources extracted

Search twenty-five

With all of the words: flood risk

With the exact phrase: managed retreat

With at least one of the words: adapt resilience

First 1,000 sources extracted

Search twenty-six

With all of the words: flood risk

With the exact phrase: managed realignment

With at least one of the words: adapt resilience

First 1,000 sources extracted

Search twenty-seven

With all of the words: flood risk

With the exact phrase: enhanced hillslope storage

With at least one of the words: adapt resilience

All sources extracted

Search twenty-eight

With all of the words: flood risk

With the exact phrase: runoff attenuation

With at least one of the words: adapt resilience

All sources extracted

Search twenty-nine

With all of the words: flood risk

With the exact phrase: deculverting

With at least one of the words: adapt resilience

All sources extracted

Search thirty

With all of the words: flood risk

With the exact phrase: slow the flow

With at least one of the words: adapt resilience

All sources extracted
